# Diagnoses and time to recovery among injured recreational runners in the RUN CLEVER trial

**DOI:** 10.1371/journal.pone.0204742

**Published:** 2018-10-12

**Authors:** Benjamin Mulvad, Rasmus Oestergaard Nielsen, Martin Lind, Daniel Ramskov

**Affiliations:** 1 Division of Sports Traumatology, Department of Orthopedics, Aarhus University Hospital, Aarhus, Denmark; 2 Section for Sports Science, Department of Public Health, Aarhus University, Aarhus, Denmark; 3 Department of Physiotherapy, University College of Northern Denmark, Aalborg, Denmark; The Ohio State University, UNITED STATES

## Abstract

**Purpose:**

The purpose of the present study was to describe the incidence proportion of different types of running-related injuries (RRI) among recreational runners and to determine their time to recovery.

**Methods:**

A sub-analysis of the injured runners included in the 839-person, 24-week randomized trial named Run Clever. During follow-up, the participants reported levels of pain in different anatomical areas on a weekly basis. In case injured, runners attended a clinical examination at a physiotherapist, who provided a diagnosis, e.g., medial tibial stress syndrome (MTSS), Achilles tendinopathy (AT), patellofemoral pain (PFP), iliotibial band syndrome (ITBS) and plantar fasciopathy (PF). The diagnose-specific injury proportions (IP) and 95% confidence intervals (CI) were calculated using descriptive statistics. The time to recovery was defined as the time from the first registration of pain until total pain relief in the same anatomical area. It was reported as medians and interquartile range (IQR) if possible.

**Results:**

A total of 140 runners were injured at least once leading to a 24-week cumulative injury proportion of 32% [95% CI: 26%; 37%]. The diagnoses with the highest incidence proportion were MTSS (IP = 16% [95% CI: 9.3%; 22.9%], AT (IP = 8.9% [95% CI: 3.6%; 14.2%], PFP (IP = 8% [95% CI: 3.0%; 13.1%]. The median time to recovery for all types of injuries was 56 days (IQR = 70 days). Diagnose-specific time-to-recoveries included 70 days (IQR = 89 days) for MTSS, 56 days (IQR = 165 days) for AT, 49 days (IQR = 63 days) for PFP.

**Conclusion:**

The most common running injuries among recreational runners were MTSS followed by AT, PFP, ITBS and PF. In total, 77 injured participants recovered their RRI and the median time to recovery for all types of injuries was 56 days and MTSS was the diagnosis with the longest median time to recovery, 70 days.

## Introduction

Running is a very popular type of exercise and the number of runners worldwide has grown over the past decades [1]. Among recreational runners, the most supported motives are to keep healthy, to maintain stamina and to reduce weight or avoid increasing their weight [2]. Running contributes to a range of health-related benefits such as lowering overall body fat, optimizing the composition of fat molecules in the blood, lowering the resting heart rate and improving the overall cardiovascular fitness [3]. In general, runners have a 25–40% reduced risk of premature mortality and live approximately 3 years longer than non-runners [4]. Owing to the health benefits and because of the considerable interest in running illuminating barriers to continued running deserves to be a key public health priority.

In Denmark, it has been estimated that 5% of the adult population, equivalent to 260,000 individuals, suffer from a running-related injury (RRI) on a yearly basis [5]. Running is hence the sports activity that contributes with most annual sports injuries in Denmark. When evaluated in a population of runners, 1-year injury incidence proportions have been reported in the range from 43.2% to 84.9% in different types of runners [6]. Running injuries were the most common reason for permanently dropping out of a running regime among males, and the third-most common reason among females according to a 10-year prospective cohort study [7]. Direct economic costs of running-related injuries range from 0.3% to 4.6% of national healthcare expenditure [8]; and some injured runners come to suffer from permanent physical disability making them unable to exercise due to pain or discomfort [9,10]. Indeed, the combination of mental and physical consequences increases the likelihood of lapsing into a sedentary lifestyle during and after injury recovery.

Running-related injuries usually occur in the lower extremity [11]. Some of the most frequent diagnoses amongst runners are patellofemoral pain (PFP), iliotibial band syndrome (ITBS) and plantar fasciosis (PF), with proportions in relation to all injuries ranging between 10–17%, 4–8%, and 5–8%, respectively [12,13]. Commonly, runners receive a referral to a physiotherapist for treatment purposes [14]. Here, many runners are concerned with the time to recovery. To provide answers, insights into diagnose-specific time-to-recoveries are needed. Unfortunately, there is a literature gap concerning the time to recovery for classical running-related injuries such as PFP, ITBS and PF. Among novice runners, the median time to recovery of all types of RRIs has been estimated to approximately 10 weeks with diagnose-specific recoveries ranging between 26 days to 174 days [13]. Still, no study has investigated the time to recovery among injured recreational runners. Consequently, the purposes of the present study were to describe the incidence proportion of different types of running-related injuries among recreational runners, engaged in the Run Clever trial [15], and to determine their time to recovery measured in days.

## Materials and methods

The present paper presents a sub-analysis of the injured participants from the Run Clever trial. The Run Clever trial was a randomized 24-week follow-up intervention study including recreational runners. The intervention was two different running schedules, the main outcome was RRIs and the participants were followed by weekly questionnaires. The two running schedules were founded on the same framework, 3 running sessions per week, and an identical 8 weeks preconditioning period followed by 16 weeks of intervention. The intervention training period was organized in cycles of 4 weeks with progression. One group, the intensity training group, had a fixed running volume but the amount of hard pace was increased during the cycles of progression. The other group, the volume training group, focused on increasing the total running volume per week but only performed at an easy or moderate pace. The original purpose was to compare overall risk of injury between progression in running intensity and running volume [15]. The Run Clever trial was approved by The Ethics Committee Northern Denmark and the Danish Data Protection Agency (N-20140069). Prior to recruitment, on January 23^rd^ 2015, the trial was registered at Clinicaltrials.gov under registration number: NCT02349373.

Healthy persons between 18 and 65 years of age were eligible for inclusion in the Run Clever trial. They had to be recreational runners free of injury in their lower extremities in the past 6 months. A recreational runner was defined as a person who had been running 1 to 3 weekly sessions for at least 6 months. The approach of recruiting participants and further criterions for inclusion or exclusion of the Run Clever trial are described in detail elsewhere [15]. The sub-sample included in the present study, were participants included in the Run Clever trial who sustained at least one RRI during the follow-up period.

At baseline, each participant was provided access to an internet-based training diary. After being registered in the diary, the participants received weekly automated e-mails including a link to an online questionnaire on injury-related pain. The questionnaire contained questions regarding symptoms of overuse or injuries based on the Oslo Sports Trauma Research Center Questionnaire (OSTRC) [16]. The OSTRC was modified with two additional questions and an additional option of answers to adapt it for the Run Clever Trial. When discomfort or an injury was registered in the OSTRC questionnaire, the participant informed on their pain in different anatomical areas, and the options were the “foot”, “ankle”, “front of lower leg”, “calf”, “knee”, “thigh”, “hamstrings”, “groin”, “glutes”, “hip” and “lower back”. The questionnaires were distributed as e-mails every Sunday to the participants’ e-mail address. The participants had to complete it whether or not suffering an injury, hereby getting information of any experienced pain the previous week. In case no response was received during the Sunday, a reminder e-mail was sent to the participant the following Monday (the day after).

In line with most recent scientific work, a RRI was defined as any physical pain or complaints from muscles, joints, bones or tendons of the lower extremities or back as a result of running [17]. It had to reduce the training performance such as distance, frequency, intensity or pace for at least 7 days [18]. When a participant reported a RRI via the weekly injury-questionnaire, an appointment with a certified physiotherapist, who was part of a study-specific diagnostic team, was made. The physiotherapist performed the clinical examinations in their respective clinics, generally within a week, and used a standardized examination procedure [13]. The physiotherapist made the standardized examination of the foot, ankle, lower leg, knee, thigh, hip or back and compared their findings with standardized, non-validated diagnostic criterions for different diagnoses [13]. The diagnosis was based on the medical history and objective findings. When the physiotherapist had completed an examination, the diagnosis (e.g., medial tibias stress syndrome (MTSS), Achilles tendinopathy (AT)) and date of examination was registered and reported to the database. No treatment or plans of rehabilitation was delivered, only a few pieces of advice at the most. However, the participant was allowed to search for treatment and receive treatment elsewhere.

The definition of time to recovery was based on the responses in the weekly OSTRC-scores on pain as well as the diagnostic examination by the physiotherapist. The date of examination and diagnosis provided by the physiotherapist were compared to the responses from the weekly OSTRC-scores to identify if pain reported via the OSTRC in the affected anatomical site corresponded with the anatomical location of the diagnosis provided by the physiotherapists. Based on this, the time to recovery was defined as the time from the first registered pain in a specific anatomical area until total pain relief in the same anatomical area. Date of recovery was defined as the date total pain relief occurred, which, then, was followed by at least three weeks without pain in the relevant anatomical site. If a participant was pain-free for a week but reported pain the following two weeks in the same anatomical location, the participant was still classified as being injured. However, if new pain arose in the same anatomical site after three weeks without pain, it was considered as a new injury. If a participant sustained two different RRIs or more during the follow-up period, only the first injury was included in the analysis.

The injured runners were excluded from the analyses on time to recovery if they did not meet the following eligibility criteria: (i) the injury had to recover before at end of 24-week follow-up, (ii) they had to answer at least ten of the weekly administered questionnaires, (iii) their pain had to be registered in the same anatomical location as the one registered by the physiotherapist, (iv) they needed to register pain (e.g., in some cases, no pain was registered at all), (v) they had to register a date of injury occurrence or (vi) the time to recovery had to be plausible compared to the diagnosis (e.g., we found pain for one week following a broken leg unreliable).

The Kaplan-Meier estimator was used to calculate the proportion of injury-free Run Clever participants as a function of weeks. As these methods takes into account censoring, the proportion of injured participants after 24-week follow-up is not number of injured runners divided by the total sample size as the latter approach assumes complete follow-up for all runners. Data on time to recovery was evaluated using histograms and 95% prediction intervals to decide if it was normally distributed. As this was not the case, non-parametric statistics were used to present time to recovery as medians and IQRs. At least five recovered injuries were required to include these calculations. The data-management and analyses presented are performed using STATA/SE version 14 and Microsoft Excel 2010.

## Results

**Fig 1:**
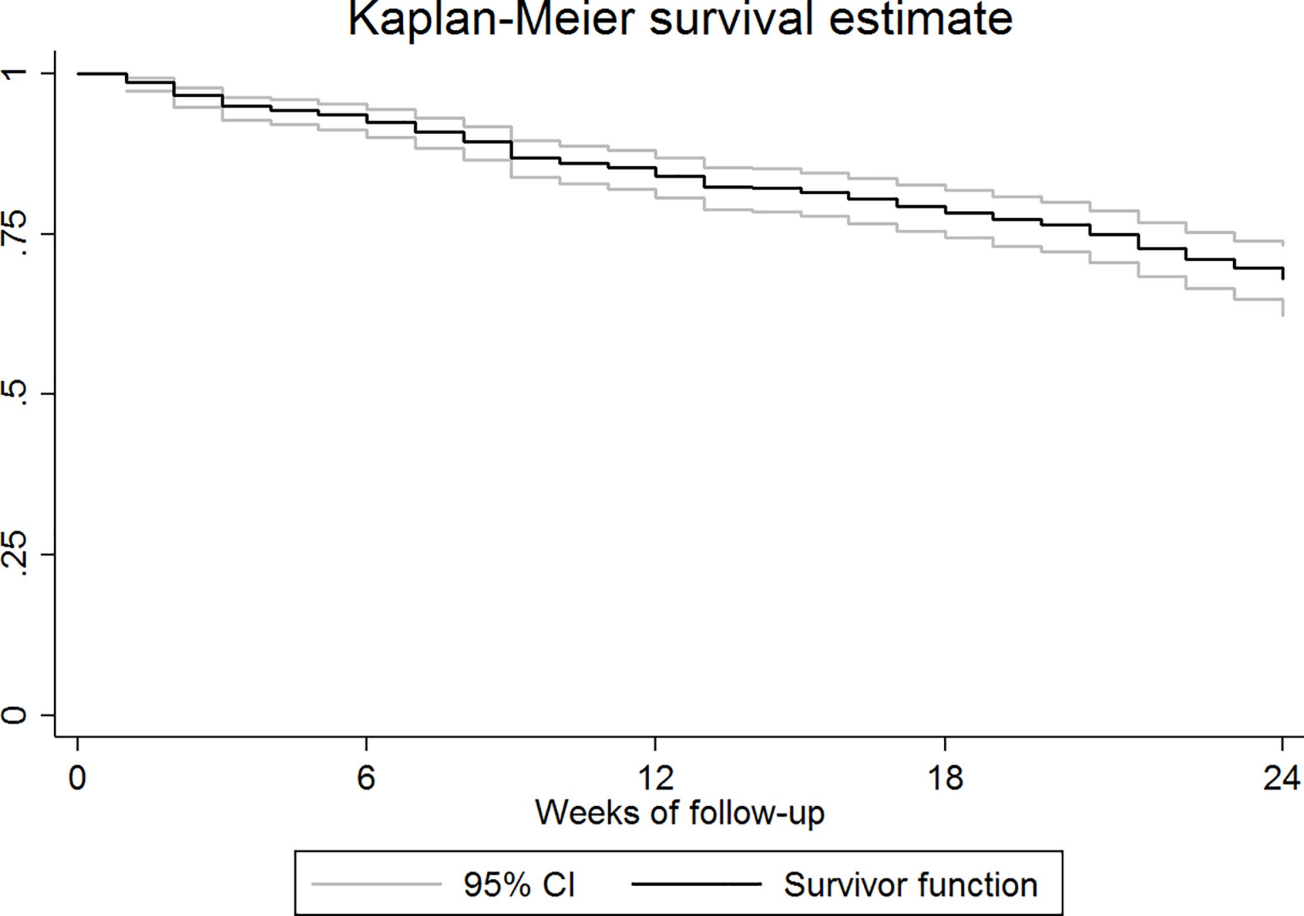
Kaplan-Meier graph. Kaplan-Meier graph visualizing the proportion of injury-free runners as a function of follow-up time. The results revealed 32% [95% CI: 26; 37] of the runners sustained injury over the 24 weeks.

**Fig 2:**
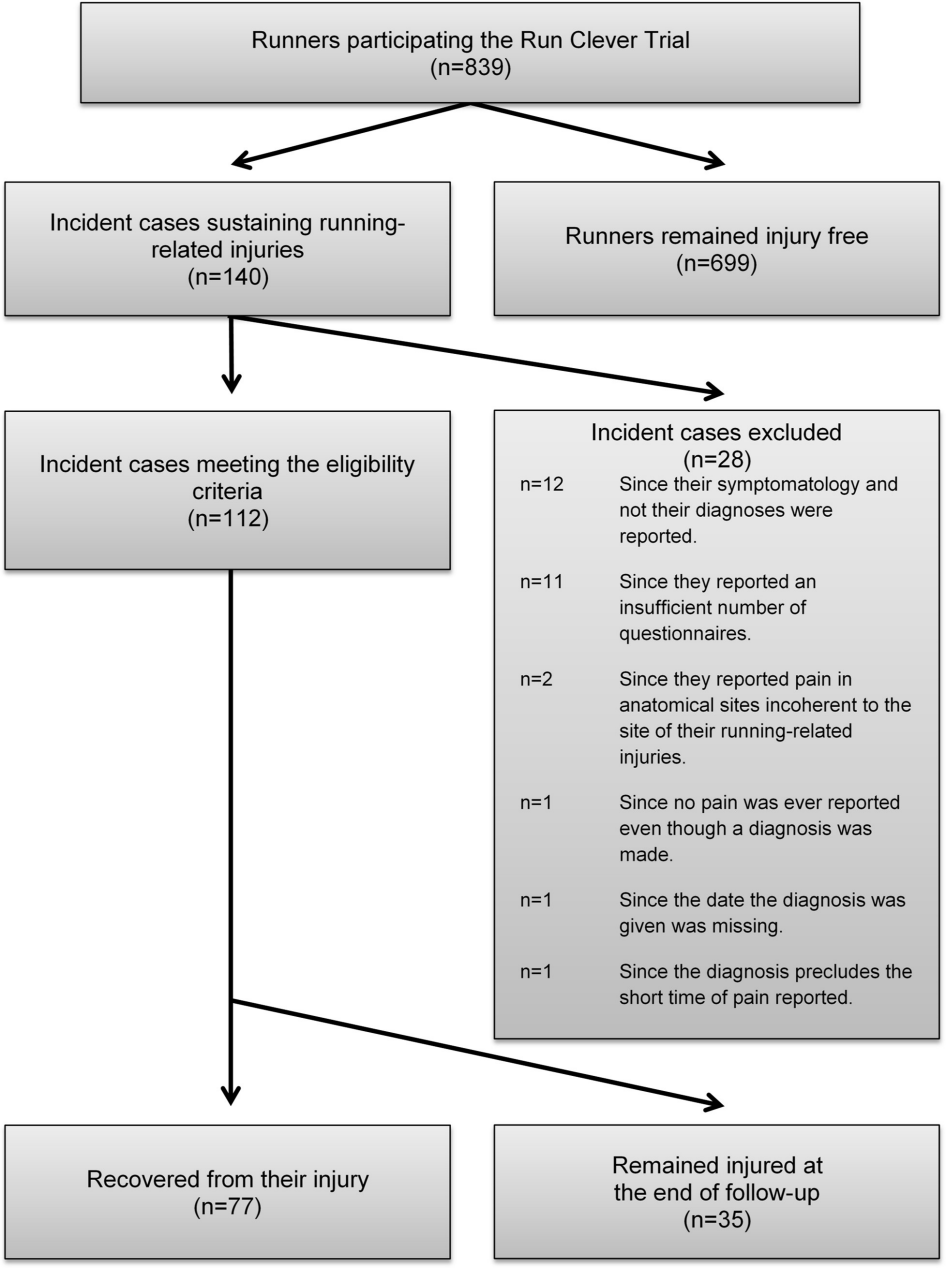
Flowchart. Flowchart visualizing the flow of runners sustaining injuries during the Run Clever trial.

A total of 839 runners participated the Run Clever Trial of whom 521 (62%) were female and 318 (38%) were male. The mean age was 39.2 (±10.0) years. 140 sustained at least one RRI during the follow-up period. A Kaplan-Meier graph visualizing the proportion of injury-free runners as a function of follow-up time is presented in Fig 1 showing that 32% [95% CI: 26; 37] of the population sustain injury over the 24 weeks. Of these, 28 injured runners were excluded since they did not meet the requirements for inclusion to the analyses (Fig 2). Among the remaining 112 injured runners, 82 (73%) were female and 30 (27%) were male, and their mean age was 41.4 years (minimum: 21 years, maximum: 63 years). A total of 1225 injury questionnaires were distributed to injured participants of which 1064 (87%) were returned successfully.

The most common RRI was MTSS reported among 18 incident cases (16% [95% CI: 9.3; 22.9]). This was followed by AT (n = 10; 8.9% [95% CI: 3.6; 14.2]), PFP (n = 9; 8% [95% CI: 3.0; 13.1]), ITBS (n = 8; 7.1% [95% CI: 2.4; 11.9] and PF (n = 8; 7.1% [95% CI: 2.4; 11.9]. In total, these five diagnoses account for 47% of the injuries. The remaining incident cases were classified within 20 other diagnosis-groups (Table 1).

**Table 1:** Incident cases, incidence proportion, and characteristics of the 25 different diagnoses of running-related injuries. Injuries are presented in descending order starting with the most frequent. n = number. The total count is presented. Non-recovered injuries are the number of RRIs still sustained at the end of follow-up. The incidence proportion of the injuries and their related confidence interval, CI 95%, are presented in percentages. The distribution of gender is presented as the number of females with each diagnosis. y = years. Furthermore, the mean age, stated in years.

Diagnosis	Incident cases, n (Non-recovered injuries, n)	Incidence proportion in % (95%CI)	Gender female (n)	Mean Age (y)
Medial tibial stress syndrome (MTSS)	18 (5)	16.07 (9.3; 22.9)	13	35
Achilles tendinopathy (AT)	10 (3)	8.93 (3.6; 14.2)	7	43
Patellofemoral pain (PFP)	9 (2)	8.04 (3.0; 13.1)	8	37
Iliotibial band syndrome (ITB)	8 (2)	7.14 (2.4; 11.9)	8	34
Plantar fasciopathy (PF)	8 (3)	7.14 (2.4; 11.9)	5	43
Gastrocnemius injury	8 (1)	7.14 (2.4; 11.9)	2	49
Gluteus medius tendinopathy	7 (2)	6.25 (1.8; 10.7)	7	42
Medial meniscus injury	7 (4)	6.25 (1.8; 10.7)	6	47
Hamstring injury	6 (2)	5.36 (1.2; 9.5)	6	37
Soleus injury	5 (0)	4.46 (0.6; 8.3)	1	47
Ankle distortion	3 (1)	2.68	3	32
Greater Trochanter Bursitis	3 (1)	2.68	3	46
Patellar tendinopathy	3 (1)	2.68	2	31
Quadriceps injury	3 (1)	2.68	1	42
Psoas major injury	2 (1)	1.79	1	46
Peroneus tendinopathy	2 (2)	1.79	1	42
Pes anserine injury	2 (2)	1.79	1	52
Adductor injury	1 (0)	0.89	1	54
External coxa saltans	1 (0)	0.89	1	40
Flexor hallucis longus tendinitis	1 (0)	0.89	1	44
Hallux valgus	1 (0)	0.89	1	40
Mortons neurom	1 (0)	0.89	1	45
Sacroiliac joint injury	1 (0)	0.89	0	43
Lower back injury	1 (1)	0.89	1	49
Stress fracture collum femoris	1 (1)	0.89	1	45
**Total**	**112 (35)**	**100**	**82**	**41**

At the end of follow-up 35 participants remained injured. Therefore, a total of 77 incident cases recovered from their RRIs before the end of follow-up and were included in the analyses on time to recovery (Table 2). The overall median time to recovery was 56 days (IQR = 70) regardless the injury diagnoses. In the diagnose-specific recoveries, the shortest median time to recovery was observed among participants sustaining PF with 35 days (IQR = 70). As opposed to this, MTSS had the longest median time to recovery with 70 days (IQR = 89). Eight participants suffered two running-related injuries, and none suffered from three or more injuries during follow-up.

**Table 2:** Incident cases recovered and their time to recovery. Diagnoses related to median time to recovery presented in decreasing order. When no median time to recovery is available, number of incident cases recovered is listed in decreasing order. Only recovered injuries are included in the table and the total count of recovered RRIs is presented. Min = minimum time to recovery. Max = maximum time to recovery. Q1 = 25^th^ percentile of time to recovery. Q3 = 75^th^ percentile of time to recovery. Interquartile ranges are presented with minimum and maximum time to recovery as well as breakdown points of 25%; all numbers are represented in days. For diagnosis with only one incident case present, the time to recovery is listed in the “min” category. * = mean time (instead of median time) to recovery presented.

Diagnosis	Incident cases recovered, n	Median time to recovery in days	Min	Q1	Q3	Max
Medial meniscus injury*	3	89	70			105
Hamstring injury*	4	74	14			140
Medial tibial stress syndrome (MTSS)	13	70	21	37	126	238
Gluteus medius tendinopathy	5	56	42	42	84	91
Iliotibial band syndrome (ITB)	6	56	14	39	105	168
Achilles tendinopathy (AT)	7	56	7	42	207	245
Patellofemoral pain (PFP)	7	49	14	28	91	119
Soleus injury	5	49	14	42	70	70
Gastrocnemius injury	7	49	7	10,5	70	91
Plantar faschiopathy (PF)	5	35	35	35	105	301
Ankle distortion	2		21			28
Quadriceps injury	2		21			70
Greater Trochanter Bursitis	2		35			70
Patellar tendinopathy	2		35			133
Psoas major injury	1		7			
Sacroiliac joint injury	1		28			
Flexor hallucis longus tendinitis	1		42			
External Coxa saltans	1		56			
Hallux valgus	1		98			
Mortons neurom	1		133			
Adductor injury	1		154			
Peroneus tendinopathy	0					
Pes anserine injury	0					
Lower back injury	0					
Stress fracture collum femoris	0					
**Total**	**77**	**56**	**7**	**35**	**105**	**301**

## Discussion

During the 24-week follow-up in the Run Clever trial, 32% of the recreational runners sustained at least one RRI. Compared with previous research this seems similar to the incidence proportion 25.9% of the novice runners in a study by Buist et al. suffering from a RRI during the 8-week observation period [19]. Moreover, Taunton et al. found an incidence proportion of RRI to be 29.5% during the 13-week training protocol before the Vancouver Sun Run [20]. Finally, in a systematic review on injuries among different types of runners, incidence proportions of RRIs were reported in the range between 20% to 80% [6]. However, these differences should be interpreted with caution because of different injury definitions and different durations of follow-up across studies. The overall median time to recovery across RRI diagnoses was 56 days among the recreational runners analyzed. Previously, the median time to recovery among novice runners has been found to exceed 70 days [13].

MTSS was the RRI diagnosis with the highest incidence proportion followed by AT, PFP, ITBS, and PF. Interestingly, these diagnoses are also among the five most common diagnoses found in previous research [12, 13, 21]. Collectively, the five diagnoses accounted for almost half the injuries sustained (47%) in the present study. This is also similar to previous studies revealing these injuries to target 42.6%, 51.8% and 41% of the injured runners, respectively [12,13,21]. Consequently, across various studies it is not uncommon that almost half the RRIs are distributed between these five diagnoses. The RRI diagnosis with the longest recovery time was medial meniscus injury followed by hamstring injury. However, the most incident RRI diagnoses, MTSS, AT, PFP, ITBS and PF in the present study were also among the top 10 RRI with the longest recovery time.

A strength of the present study is the weekly status updates, which reduced the risk of recall bias and information problems. Furthermore, the diagnostic approach, encompassing a standardized physical examination performed by a study-specific diagnostic team of physiotherapists, ensured a greater certainty of accurate injury diagnosis as well as exact date of injury occurrence.

Very few comparable studies exist, but an interesting finding is the time to recovery among the recreational runners sustaining MTSS of median 70 days. Since, comparable recovery times of 72 days in a study on novice runners [13], 82 days among infantry recruits in the British army [22], and 58 days among 15 military recruits from the Royal Dutch army [23] have been reported.

However, differences in the populations investigated and definitions of recovery should be considered. The main reason for the discrepancy in definition of injury recovery between the present study and the previous DANORUN study also including runners by Nielsen et. al, stems from the different ways the data was collected [13]. The electronical database facilitated more frequent and standardized follow-up in the Run Clever trial allowing for a better evaluation of the levels of pain and symptoms. Furthermore, the altered definition of injury recovery enabled to avoid runners being labeled injury-free though they participated in running with injuries.

Still, some limitations exist. Firstly, in total, 35 participants did not recover their RRIs before the end of follow-up. For instance, only 3 of the 7 runners with medial meniscal injured recovered. For these three runners, the median time-to-recovery was 89 days. However, if the remaining four runners had been followed until recovery it is likely the case that the median time-to-recovery would have been longer. This underestimation targets many diagnose-specific recovery-times as the proportion of individuals with medial meniscus injury, MTSS, ITB and AT who became injury-free ranged from 42.3%–70%, respectively. Further, comparing time to recovery in the current study, to recovery times from the study by Nielsen et al. [13] a considerable difference in the diagnoses specific maximum values reported becomes evident. A reason for this may be the definition of recovery in the current study including a margin of three consecutive pain-free weeks was different that the one used in other studies. Consequently, an extended follow-up time would have been preferred to reduce the loss of data.

Secondly, the diagnostic approach was standardized to reduce the risk of subjective information bias regarding the diagnosing for which reason every injury was diagnosed on the basis of a physical examination and the injured runner’s anamnesis. Making a diagnosis adhering to the guidelines was not always possible, which makes the objectivity less solid. Thirdly, the definition of recovery is complex. The RRI was deemed to be recovered after three successive weeks without any pain during running in the related anatomical site, but no physical examination or test was performed to make sure full recovery was attained. Moreover, the experience of pain might be diverse in different injuries so that the three-week distinction might be undiscriminating.

Despite various limitations in the present study, the results may be of interest for both researchers and clinicians dealing with RRIs. The present study is a prospective analysis of data obtained from the Run Clever trial in which information on new injury onset and exact diagnosing were very important and as proper as possible. However, a major drawback was the lack of continually follow-up on the accuracy on the information submitted by the injured participants.

## Conclusion

The cumulative incidence proportion of injured participants in the Run Clever trial was 32%. The injuries were classified across 25 different diagnoses with MTSS, AT, PFP, ITBS and PF as the most frequent ones. Altogether, these five diagnoses accounted for 47% of all injuries. The median time to recovery for all types of injuries was 56 days. MTSS was the diagnosis with the longest median time to recovery of 70 days.

## Supporting information

S1 Dataset A STATA.dta file. (DTA)
